# Using mixed methods evaluation to assess the feasibility of online clinical training in evidence based interventions: a case study of cognitive behavioural treatment for low back pain

**DOI:** 10.1186/s12909-016-0683-4

**Published:** 2016-06-18

**Authors:** Helen Richmond, Amanda M. Hall, Zara Hansen, Esther Williamson, David Davies, Sarah E. Lamb

**Affiliations:** Centre for Rehabilitation Research in Oxford, Nuffield Department of Orthopaedics, Rheumatology and Musculoskeletal Sciences, University of Oxford, Oxford, UK; Warwick Clinical Trials Unit, Warwick Medical School, University of Warwick, Coventry, UK; The George Institute for Global Health, University of Oxford, Oxford, UK

**Keywords:** Low back pain, Cognitive behavioural, Online training, Implementation, Dissemination, Physiotherapy, Mixed methods, E-learning, Evidence-based practice, Psychological

## Abstract

**Background:**

Cognitive behavioural (CB) approaches are effective in the management of non-specific low back pain (LBP). We developed the CB Back Skills Training programme (BeST) and previously provided evidence of clinical and cost effectiveness in a large pragmatic trial. However, practice change is challenged by a lack of treatment guidance and training for clinicians. We aimed to explore the feasibility and acceptability of an online programme (iBeST) for providing training in a CB approach.

**Methods:**

This mixed methods study comprised an individually randomised controlled trial of 35 physiotherapists and an interview study of 8 physiotherapists. Participants were recruited from 8 National Health Service departments in England and allocated by a computer generated randomisation list to receive iBeST (*n* = 16) or a face-to-face workshop (*n* = 19). Knowledge (of a CB approach), clinical skills (unblinded assessment of CB skills in practice), self-efficacy (reported confidence in using new skills), attitudes (towards LBP management), and satisfaction were assessed after training. Engagement with iBeST was assessed with user analytics. Interviews explored acceptability and experiences with iBeST. Data sets were analysed independently and jointly interpreted.

**Results:**

Fifteen (94 %) participants in the iBeST group and 16 (84 %) participants in the workshop group provided data immediately after training. We observed similar scores on knowledge (MD (95 % CI): 0.97 (−1.33, 3.26)), and self-efficacy to deliver the majority of the programme (MD (95 % CI) 0.25 (−1.7; 0.7)). However, the workshop group showed greater reduction in biomedical attitudes to LBP management (MD (95 % CI): −7.43 (−10.97, −3.89)). Clinical skills were assessed in 5 (33 %) iBeST participants and 7 (38 %) workshop participants within 6 months of training and were similar between groups (MD (95 % CI): 0.17(−0.2; 0.54)). Interviews highlighted that while initially sceptical, participants found iBeST acceptable. A number of strategies were identified to enhance future versions of iBeST such as including more skills practice.

**Conclusions:**

Combined quantitative and qualitative data indicated that online training was an acceptable and promising method for providing training in an evidence based complex intervention. With future enhancement, the potential reach of this training method may facilitate evidence-based practice through large scale upskilling of the workforce.

**Trial registration:**

Current Controlled Trials ISRCTN82203145 (registered prospectively on 03.09.2012).

**Electronic supplementary material:**

The online version of this article (doi:10.1186/s12909-016-0683-4) contains supplementary material, which is available to authorized users.

## Background

Low back pain (LBP) is one of the largest challenges facing public health systems in the western world [1]. Cognitive behavioural (CB) approaches are recommended for the management of non-specific LBP [2]. The 2009 National Institute for Health and Care Excellence (NICE) guideline for non-specific LBP [3] fell short of making strong recommendations for a CB approach due to a lack of evidence. However, since the publication of the NICE guidance an additional eight randomised controlled trials (RCTs) have reported effect sizes that support use of a CB approach to manage LBP [4]. Thus, it is now widely accepted that LBP should be managed with a programme that utilises a CB approach. Moreover, the UK National Spinal Taskforce has identified the provision of such programmes to be the most serious gap in current LBP service provision that should be urgently addressed [5].

Implementing new evidence-based approaches to care often requires clinicians to learn new skills and change their consultation behaviours [6, 7]. For allied health professionals, this means learning how to use a CB approach in clinical practice such as the optimal dosage, delivery mode, and combination of treatment components. Addressing this, the CB Back Skills Training programme (BeST) follows a set structure and provides clinicians with detailed guidance in a manual about how to deliver a group-based programme to patients [8–11]. BeST is underpinned by the broad CB approach literature and provides an evidence based approach in sufficient detail to allow implementation [12]. In the original pragmatic trial, we provided a 2-day face-to-face training workshop, along with a detailed manual and materials to support the programme sessions. We are now seeking to achieve wide-scale implementation beyond the pragmatic trial in which it was initially evaluated.

This translation and implementation of research products into the clinical setting provides a number of challenges for researchers. Research teams are often small, and providing an implementation strategy scalable to national and international demand, without the financial underpinning provided by research grants, is almost impossible. Hence, we have developed an online training programme (iBeST) to disseminate BeST materials and provide training in a CB approach. This scalable method places less demand on resources, is not geographically constrained, and offers greater flexibility to the learner [13]. However, due to a paucity of research on the use of online methods for delivering training in psychologically informed treatments to allied health professionals, the feasibility and acceptability of this method needed to be explored as part of a staged implementation plan toward providing national and international access to the BeST training and intervention materials.

### Aim/Objectives

In line with the Medical Research Council’s guidance for the development of complex interventions, this study aimed to explore the feasibility and acceptability of training physiotherapists with iBeST prior to a larger scale evaluation of effectiveness [14]. Therefore, using a face-to-face workshop as a gold-standard reference, we wanted to explore the potential effect of iBeST on learning outcomes, as well as ascertaining physiotherapists’ acceptance of and satisfaction with iBeST. Secondary objectives included examining physiotherapists’ use of and experiences with iBeST and monitoring uptake of a CB approach (BeST) in clinical practice.

## Methods

### Design

A mixed methods evaluation, consisting of an exploratory randomised controlled trial and individual semi-structured interviews, was conducted between May 2013 and December 2013. Ethical and governance approval was granted from the University of Warwick’s Biomedical and Scientific Research Ethics Committee (reference number 244-10-2012).

### Participants

Participants were volunteers that responded to a substantial email request distributed through research network mailing lists and managerial staff in NHS Trusts. While the BeST programme can be delivered by nursing, allied health, and psychological professions, we concentrated on physiotherapists since they provide the majority of LBP care in the UK NHS [15]. Eligible participants were NHS physiotherapists managing a LBP patient caseload, based in Warwickshire or neighbouring counties, with access to the Internet. We did not exclude participants based on any prior training or current practice behaviours.

### Brief description of the BeST programme

The BeST programme is underpinned by a CB approach and consists of an initial individual session of 60 min, and six group sessions of 90 min with 5 to 10 patients per group. It uses patient-specific needs to guide goal setting and treatment planning. It provides education about persistent pain, the importance of regular exercise, the relationship between activity levels and pain, and the role of unhelpful thoughts and behaviours in the maintenance of LBP. It teaches patients a range of skills including problem solving, goal setting, baseline setting, relaxation, thought challenging, planning for flare ups, activity pacing, and activity progression. Additionally, patients collaboratively negotiate a tailored exercise programme to do at home. Each group session follows the same structure, and begins with agenda setting and a review of homework, covers 1–2 session topics, has a 10 min break halfway through, and ends with homework setting.

## The randomised controlled trial (Current controlled trials ISRCTN82203145)

Participants giving their informed consent were randomised to receive iBeST or a face-to-face workshop according to a computer generated random number sequence that was stratified by centre. The allocation sequence was concealed in sealed opaque envelopes and was held offsite and administered by an external, independent researcher. Participant blinding was not possible due to the nature of the interventions.

### Description of interventions

Apart from the mode of delivery, we took care to ensure that both training methods were the same, including: the knowledge content, skills training, and training resources (therapist manual, session narratives and crib sheets, patient workbook, and additional information sources).

### Face-to-face workshop

Participants randomised to the workshop attended for two days of face-to-face training, replicated from the original BeST trial [9], at the University of Warwick in May 2013. In brief, the training consisted of PowerPoint presentations, video clips, role-play scenarios, discussion and feedback. Participants were issued with a training pack that contained all slides, the therapist manual, and patient workbook. They also had access to a website where they could download additional paperwork only.

### iBeST

Development: iBeST content was produced in Adobe Captivate and hosted within the virtual learning environment, Moodle (Moodle.com, Perth, Australia). Constructivism, which states that learners actively construct knowledge through gaining understanding, and that new knowledge can only be built upon current understanding [16], was the predominant theory underpinning the organisation of online content. Course features included self-directed reading, reflective practice, skill rehearsal, multiple-choice questions, formative tests with feedback, interactive exercises, a discussion forum, and multimedia. There were 10 core modules to complete.

Procedures: The course was designed to take an equivalent learning time to that of the workshop (10 h). Participants could pace the course to their own preference, and did not have to complete it over a set time (i.e., two days). Participants were emailed a username, password, and start-up guide. We requested that they completed the programme within 6-weeks, which was accessible 24 h/day. Following course completion, participants maintained programme access.

We encouraged participants in both groups to implement the BeST programme after completing the training; however, this was not enforced.

### Outcome measures

#### Demographics

We collected baseline demographics, including gender, job title, time worked in profession, age range, degree of experience with a CB approach, and training preference before randomisation.

### Outcome measure timings

All outcomes, excluding the assessment of clinical skills, were collected from participants immediately after they had completed the training. For those in the workshop comparison, this meant completing questionnaires before leaving the workshop venue. For participants in the iBeST group, this meant completing the questionnaires online within one week of finishing all modules. Since an assessment of clinical skills required the participants to set-up the intervention in their clinical practice, we allowed a 6-month time frame from completing the training within which to set up the intervention. Thus, the exact time of assessment for this outcome post-training was variable for each participant.

### Learning outcomes

We aimed to assess two aspects of knowledge: (i) theoretical Knowledge of CB approach and (ii) procedural knowledge of how to deliver a CB approach in clinical practice. Since no validated or specific questionnaire was available to assess this, we developed a bespoke multiple-choice questionnaire (scale 0–31, lower score indicates lower knowledge). Questions to assess theoretical knowledge of a CB approach were derived from the background teaching of the training and focused on the CB model and its applications. To assess procedural knowledge, questions concerned how aspects of a CB approach could be delivered in relation to the BeST programme.

For participants who delivered a CB approach in clinical practice, we audio recorded a single treatment session and assessed clinical skills with the 15-item Cognitive Therapy Scale-Revised-Pain (CTS-R-Pain; scale 0–6, lower score indicates lower skill level) [17]. This tool has been specifically modified to measure competency in the use of a CB approach among non-psychology specialists. Hansen et al. [18] found the tool to have high internal consistency (Cronbachs α = 0.99) and good inter and intra-rater reliability (intra-class correlation coefficient for intra-rater reliability = 0.92 (0.79; 0.97). One rater (unblinded) with training in a CB approach (HR) scored all recordings and a senior blinded rater with comprehensive experience in a CB approach (ZH) doubly assessed 25 % of recordings.

In addition to assessing knowledge and clinical skills, we assessed participants’ attitudes, self-efficacy, and satisfaction as recommended in Kirkpatrick’s training evaluation model [19–21]. Attitudes and beliefs towards the management of persistent LBP were assessed pre and post training with the 31-item Pain Attitudes and Beliefs Scale for Physiotherapists (PABS-PT). The use of a CB approach aligns itself with psychosocial attitudes and beliefs towards the management of LBP. The PABS-PT is well validated and has two subscales: biomedical (includes 14 items) and psychosocial (includes 6 items) which are rated with a Likert scale (range 1–5) [22]. A lower score on the biomedical scale indicates lower biomedical orientation and a lower score on the psychosocial scale indicates lower psychosocial orientation. Self-efficacy to deliver the (i) single BeST individual and (ii) six group sessions was assessed with two Likert scales (scale 0–10, lower score indicates lower self-efficacy). The six group sessions formed the majority of the BeST programme. We assessed training satisfaction (acceptability) with a custom single-item measure (scale: 1–5, lower score indicates lower satisfaction).

To assess therapist’s engagement with the iBeST programme we examined the number of training slides visited, the time spent on each slide, and the number of resources (links/downloads) accessed (score range: 1–3; 3 indicated higher engagement). To interpret the scores we labelled participants who scored in the upper tertile of engagement scores as having higher engagement. Use of the BeST programme in practice was assessed by whether the participant implemented the BeST programme within a 6 month timeframe in their clinical setting.

### Sample size

This study aimed to explore the feasibility and acceptability of iBeST. Sample sizes of 24–50 participants have been recommended for assessing the feasibility of an intervention [23, 24]. Therefore, we considered a sample size of 30 participants to be sufficient to explore the feasibility and the potential effect of iBeST on learning outcomes. Allowing for a 15 % drop out rate, a total of 35 physiotherapists were recruited.

### Statistical analysis

We used the equivalent of intention to treat analysis, including all eligible randomised participants who provided follow up data. For continuous outcomes, between group differences were explored using the Students *t*-test [25]. Mean change in PABS-PT scores were adjusted to account for baseline score using an analysis of covariance (ANCOVA) [26]. Effect sizes were calculated with Hedges’ g with adjustment for small sample bias. Categorical outcomes were analysed using Fishers exact test for association [26]. Statistical significance was set at 0.05 and all effect estimates were provided with 95 % confidence intervals [25]. Descriptive statistics were used to report learner analytics.

### Exploratory analyses

Evidence suggests that training preference may impact on user engagement and satisfaction [27]. Therefore, in a pre-specified sub group analysis we stratified our results according to training preference (received preference/had no preference versus did not receive preference). Additionally, we explored whether engagement with iBeST impacted on learning (higher engagement versus all others). Analyses were summarised descriptively (mean and standard deviation) and were not subject to statistical testing.

## The interview study

Face-to-face semi-structured interviews were completed with iBeST-trained physiotherapists to explore their experiences of using iBeST. We aimed to interview all participants trained with iBeST in the RCT (=16).

### The interview guide

The initial interview guide was informed by two recent systematic reviews [28, 29] and a theoretical framework of online learning strategies to enhance health care professionals learning experience [30]. The guide was modified as the analysis of interviews progressed to ensure it was responsive to the data and to enable exploration of emerging themes. One researcher (HR) completed all interviews, which were audiotaped, transcribed verbatim and anonymised for analysis.

### Interview data analysis

Interview transcripts were analysed using an inductive thematic analysis drawing upon constructivist grounded theory (open coding and constant comparison). Open coding identified the range of concepts used by participants and resulted in categories, ensuring identified themes were grounded in the data [31]. Constant comparison and close attention to deviant cases facilitated assessment of relationships between categories [32]. Transcripts were coded by the lead author (HR) and emerging codes were discussed with a senior researcher (EW), who independently coded three transcripts.

During analysis it became apparent that data relating to theme three (training impact) were congruent with Kirkpatrick’s training evaluation model, so we considered and contextualised this theme in relation to this model [19].

#### Data integration

Integration of both quantitative and qualitative data is often cited as the heart of mixed methods research [33]. Both data sets were analysed concurrently, independently of each other, and were jointly interpreted. After assessing complementarity of the data sets, qualitative data was used to illuminate and expand upon quantitative outcomes to achieve a more comprehensive and meaningful understanding of the feasibility and acceptability of iBeST [34].

## Results

### RCT findings

**Fig. 1:**
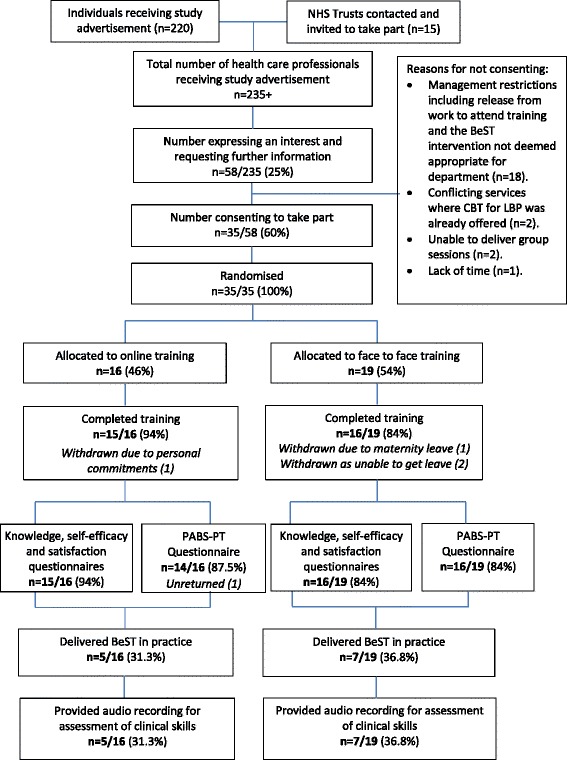
Randomised controlled trial participant flow

A minimum of 235 health care professionals received the study advertisement through research network mailing lists (*n* = 220), and direct contact with NHS Trust physiotherapy managers (*n* = 15). From which, 58 responded and 35 were recruited into this study from 8 NHS Hospital Trusts, and were subsequently randomised to receive iBeST (16 therapists) or a face-to-face workshop (19 therapists). Participant flow is provided in Fig. 1. Eighty-nine percent of participants completed training and provided follow up data (*n* = 31; iBeST: 15/16; workshop: 16/19). Twelve (34 %) therapists implemented the BeST programme in practice and thus, were able to provide data for the clinical skills assessment.

While the workshop group had a higher proportion of males, all remaining baseline characteristics from the randomised sample were broadly similar (Table 1). Although preferences for face-to-face training were well matched across the groups, there were small differences in those with no preference and with an online preference. However, the overall small sample size makes these difficult to evaluate. Additionally, due to the small numbers in some of the cells, for example ‘Training in CBT (answer: no)’, a difference of one person between the groups makes the proportion appear higher.

**Table 1:** Baseline characteristics of participants by allocation

Category	Workshop	iBeST	Total
	*n* = 19	*n* = 16	*n* = 35
Sex			
Male, n. (%)	7 (37)	3 (19)	10 (29)
Female, n. (%)	12 (63)	13 (81)	25 (71)
Age (years)			
18–25, n (%)	1 (5)	2 (12.5)	3 (8.5)
26–35, n (%)	11 (58)	7 (44)	18 (51)
36–45, n (%)	5 (27)	4 (25)	9 (26)
46–55, n (%)	1 (55)	2 (12.5)	3 (8.5)
56–65, n (%)	1 (5)	1 (6)	2 (6)
Years worked in profession			
No.	19	16	35
M (SD)	10.08 (8.045)	14.25 (10.872)	11.99 (9.532)
Median	7	10	8
Range	2–30	2–35	2–35
Training in CBT			
Yes, n (%)	16 (84)	12 (75)	28 (80.0)
Formal, n (%)	5 (31)	6 (50)	11 (39)
Informal, n (%)	11 (69)	6 (50)	17 (61)
No, n (%)	3 (16)	4 (25)	7 (20.0)
Access to a computer			
Daily, n (%)	18 (95)	12 (75)	30 (86)
2–3 times/week, n (%)	0 (0)	2 (12.5)	2 (5.7)
3–4 times/week, n (%)	0 (0)	2 (12.5)	2 (5.7)
4–5 times/week, n (%)	1 (5)	0 (0)	1 (3)
Location of access			
Work only, n (%)	7 (37)	6 (37.5)	13 (37.1)
Work and home, n (%)	12 (63)	10 (62.5)	22 (62.9)
Home only, n (%)	0 (0)	0 (0)	0 (0)
Training Preference			
None, n (%)	9 (47)	5 (31)	14 (40)
Online, n (%)	2 (11)	4 (25)	6 (17)
Face to face, n (%)	8 (42)	7 (44)	15 (43)
PABS-PT Factor 1 (biomedical attitudes and beliefs)			
No.	19	16	35
M (SD)	32.05 (7.314)	28.75 (4.374)	30.54 (6.289)
Median	32	30	30
Range	20–49	17–34	17–49
PABS-PT Factor 2 (psychosocial attitudes and beliefs)			
No.	19	16	35
M (SD)	23.26 (3.347)	23.31 (2.869)	23.29 (3.092)
Median	22	23.5	23
Range	17-29	18-28	17-29

*PABS-PT* psychosocial attitudes and beliefs scale for physiotherapists

Outcomes are reported in Table 2 and summarised below.

**Table 2:** Mean difference in outcome measures between groups

Outcome measure	Workshop		iBeST		Mean difference	P-value	Effect size^a^
	mean (SD)	N	mean (SD)	N	(95 % CI)		(95 % CI)
Cognitive Therapy Scale-Revised-Pain (CTS-R Pain)^c^	2.08 (0.33)	7	1.90 (0.18)	5	0.17 (−0.2; 0.54)	0.32	−0.59
							(−1.78; 0.59)
Knowledge^c^	25.53 (3.27)	16	26.5 (2.96)	15	0.97 (−1.33; 3.26)	0.4	0.30
							(−0.41; 1.01)
Change in Psychosocial Attitudes and Beliefs Scale for Physiotherapists (PABS-PT): biomedical subscale^b^	−8.1 (4.07)	16	−0.67 (4.87)	14	−7.43 (−10.97; −3.89)	< 0.01	−1.62
							(−2.46; −0.78)
Change in PABS-PT: psychosocial subscale^c^	2.83 (5.67)	16	−0.52 (3.52)	14	3.35 (−0.19; 6.89)	0.06	−0.68
							(−1.42; 0.06)
Self-efficacy: individual assessment^c^	7.38 (1.58)	16	5.65 (1.95)	15	1.73 (0.43; 3.03)	0.01	−0.95
							(−1.70; −0.20)
Self-efficacy: group sessions^c^	6.45 (2.50)	16	6.3 (1.75)	15	0.25 (−1.7; 0.7)	0.34	−0.07
							(−0.77; 0.64)
Satisfaction^c^	4.69 (0.48)	16	3.73 (0.70)	15	0.95 (0.52; 1.39)	< 0.01	−1.57
							(−2.39; −0.75)

^a^Negative effect size favours face-to-face workshop; ^b^A decrease in or lower score indicates an improvement on this variable; ^c^A increase in or higher score indicates an improvement on this variable

#### Knowledge

We found no statistically significant difference between training groups on knowledge (Mean Difference (MD) 0.97, 95 % CI −1.33, 3.26)). Scores ranged from 19.5 to 30 in the iBeST group, and from 20 to 30.5 in the workshop group.

#### Clinical skills

As shown in Fig. 1, this outcome could only be assessed in the participants that delivered the BeST programme in clinical practice. Of these participants, we found no statistically significant difference between training groups (MD 0.17, 95 % CI −0.2, 0.54).

#### Self-efficacy

Table 2 shows that participants trained with iBeST reported lower self-efficacy to deliver the single BeST individual assessment session (MD 1.73, 95 % CI 0.43, 3.03). However, self-efficacy to deliver the majority of the BeST intervention, the six group sessions, was similar in both groups (MD 0.25, 95 % CI −1.7, 0.7). The score range for self-efficacy (individual assessment session) was reported as 4.4 to 9.7 in the workshop group, and 1.5 to 9.7 in the iBeST group.

#### PABS-PT

There was a large and statistically significant between group difference observed in the PABS-PT biomedical subscale in favour of the workshop (MD −7.43, 95 % CI −10.97, −3.89), indicating that workshop participants held less of an orientation towards a biomedical treatment approach after training. We observed a small between group difference in favour of the workshop on the PABS-PT psychosocial subscale, suggesting a greater psychosocial orientation after training, although this difference was not statistically significant (MD: 3.35, 95 % CI: −0.19, 6.89).

#### Training satisfaction

We observed a statistically significant between group difference of nearly 1-point on the 5-point scale in favour of the workshop group (MD 0.95, 95 % CI 95 % CI 0.52, 1.39). The majority of iBeST users were ‘satisfied’, and the majority of workshop participants were ‘very satisfied’.

#### Implementation

Twelve of thirty-five therapists (34 %) delivered the BeST programme in their practice (iBeST 5/19, 31 %; workshop 7/15, 37 %) at five of eight sites (62 %). Every site had participants from both training groups and we saw no significant difference in the proportion of therapists delivering the BeST programme by training method (*p* = 0.41). Participants delivering the intervention were more senior than those not delivering the intervention, being older (*p* = 0.013) and having worked for longer in their profession (*p* = 0.001; 95 % CI: −22.0; −4.0).

#### iBeST engagement

The mean time spent using the online course was 6 h and 48 min (range (hr: mm): 1: 32 to 15: 49). Overall, compliance with the online programme was high. Half the participants (*n* = 8) completed 100 % of the course. Three participants accessed less than 50 % of each module. No participants used the online discussion forum.

### Exploratory analyses

Participants allocated to their preference were the most satisfied in both training arms. Higher engagement with the course (according to the learner analytics) corresponded with higher knowledge scores (MD (95 % CI) 3.06 (0.08; 6.03)), greater self-efficacy to deliver the majority of the programme (MD (95 % CI) 1.97 (0.27; 3.67)), larger increases in PABS-PT psychosocial subscale score (MD (95 % CI) 2.26 (−1.1; 7.01), and greater decreases in PABS-PT biomedical subscale score - as desired (MD (95 % CI) -3.51 (−9.26; 2.23).

## Interview study

**Fig. 2:**
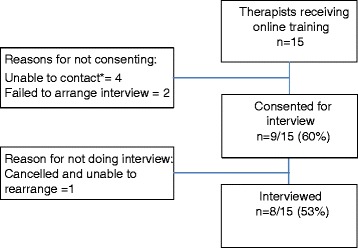
Interview study participant flow. Legend: *Attempts were made via email in the first instance and subsequently via telephone. Efforts to contact participants ceased after four attempts

The fifteen therapists from the RCT (=16) who completed iBeST were invited to take part in an interview, eight of whom consented to be interviewed. Figure 2 details participants flow through the interview study.

The sample captured a range of characteristics including age, prior CBT training, satisfaction with the training, and prior training preference (Table 3). All interviewed participants were classified as having higher engagement with iBeST and were female. Data analysis revealed three overarching themes: (1) preconceptions of online learning, (2) reflections on training experience with iBeST, and (3) impact of training with iBeST. These themes are presented in Table 4 with sub-categories and supporting participant quotes, and are described below.

**Table 3:** Interview study participant characteristics

Time	ID	208	289	243	258	337	197	257	226
Baseline	Age range (years)	26–35	56–65	36–45	26–35	46–55	36–45	36–45	46–55
	Time in profession (years)	6	35	15	8	31	21	22	31
	Preference	FP	NP	FP	FP	FP	NP	OP	FP
	Method of training	iBeST	iBeST	iBeST	iBeST	iBeST	iBeST	iBeST	iBeST
	Prior training in CBT	None	Yes	Yes	None	Yes	Yes	None	Yes
Post-training	Knowledge^a^	26	27.5	29	24.5	27	27	29.5	30
	Satisfaction	S	N	S	U	VS	S	S	S
	Self-efficacy: Assessment^a^	9.7	8.9	4.4	5.1	5.9	6.1	4	6.5
	Self-efficacy: Group^a^	8	8	8.4	7	6.5	6.5	6	7.3
	Change in PABS-PT: biomedical subscale ^b^	−1	4	−8	1	6	−5	−12	0
	Change in PABS-PT: psychosocial subscale ^a^	0	4	−2	1	−5	−2	4	0
	CTS-R-Pain score^a^	1.79	1.93	n/a	n/a	2	n/a	2.13	1.67

*FP* face-to-face, *NP* none, *OP* online, *VS* very satisfied, *S* satisfied, *N* neither, *U* unsatisfied, *PABS-PT* psychosocial attitudes and beliefs scale for physiotherapists, *CTS-R-Pain* cognitive therapy scale-revised-pain

^a^An increase in or higher score indicates an improvement on this variable

^b^A decrease in or lower score indicates an improvement on this variable

**Table 4:** Themes, sub-categories and quotes from interview participants

Theme	Sub categories	Participant quotes
Preconceptions of online learning (prior to iBeST)	Negative experience with online training	*“I don’t learn that well, especially if it’s something that I haven’t done before, just reading from a computer screen.” #208*
		*“…it is hard because you can’t really replace actually doing it, can you really, actually having it as a little role play or just questions and answer type things.” #257*
	Perceived learning style	*"…you know, the environment we work in, we don’t sit at computers all day long and we’re used to the sort of talking part of it.” #258 (learning style suits face-to-face training)*
Reflections on training experience with iBeST	Barriers to online learning	*External: “…I ended up only being able to do it at home because we couldn’t get the Google Chrome here at work at all…” #197*
		*Internal: “…in a physio department we have a patient for half an hour or an hour and you get up and, you don’t have to just sit and do one thing for 7 h and I think I’ve never worked at a computer for that length of time, so I think it just amazed me how uncomfortable it is sitting for long stretches of time.” #258*
	Facilitators to online learning	*“I think because you are seeing patients in between it made you think about how you could actually apply what you’d just been reading” #257*
Impact of the training with iBeST (after iBeST)	Participant reactions	*“I think actually it was a much better variety than I thought. I didn’t think that we would see videos. I thought it would just be words and tests really, so I think it was actually very well done.” #337*
		*“I don’t normally go home and do any work but I didn’t find it a problem going home and keeping going because it was interesting…” #257*
	Participant learning	*“…because there's one thing to say this is where pain is, what are you going to do about it? Another to say, this is what you're going to do about it - and this is how you're going to roll it out to your patients… it was just put about in a very complete way with a package to carry over.” #226*
		*“We talk about pacing in very general ways, whereas there was obviously quite specific guidelines on helping people to find their baseline and how to develop that on from their role than just generally saying don’t do everything on one go.” #257*
		*“…I think the talking part of it was the important part…and I found that really difficult to do on my own to a computer.” #258*
		*“I kind of don’t … maybe didn’t quite understand enough about today’s one about thinking.” #337*
	Participant behaviour	*“I found I was able to, as you started to think about it more, it sort of made you ask questions… talking to patients you start just bringing some of the questioning methods…” #257*
		*“…it has changed my practice - and I do talk to people differently, and try not to be so directive…” #226*

### Preconceptions of online learning (prior to iBeST)

Participants were initially sceptical that iBeST could provide the training needed to deliver the BeST programme. This scepticism arose from negative past experiences with online training, from their professed learning style, and from the perceived nature of the skills required to deliver BeST (such as needing to practice the group format). For example, prior to the training, participant #226 thought it was *“ridiculous to learn BeST online as you needed to be able to interact with people”.*

### Reflections on training experience with iBeST (during iBeST)

Therapists identified a number of barriers to engaging with iBeST that were categorised into external and internal factors. Externally, barriers included technical difficulties in gaining access to the online training programme due to out of date web browsing software on NHS Trust computers, distractions when working in the home environment, and trying to prioritise the training over other aspects of their work load. Internal barriers included sitting for long periods, self-discipline not to skip sections and ‘cheat’, lone working without the capacity for any face-to-face discussion, concentration on a computer screen, and their openness to change. For example, participant #337 noted that you could *“take quite a lot of shortcuts”* with an online course, skipping the course content and *“going straight to the test”*. Conversely, one therapist felt this training method allowed greater integration and application of learning within their clinical practice.

### Impact of iBeST training

#### Therapist reactions

The majority of participants completing iBeST (*n* = 11 of 15; 73 %) were satisfied with the training, and found it engaging. For example, participant #257 said *“I don’t normally go home and do any work but I didn’t find it a problem going home and keeping going because it was interesting.”* For one participant (#258) who was unsatisfied with the training, iBeST was unable to provide the desired level of clinical skills practice. The remaining three participants were neither satisfied nor dissatisfied with iBeST.

#### Therapist learning

Participants referred to improvements in their knowledge of persistent pain and made reference to holding a better understanding of behavioural skills, such as pacing and goal setting. For example, participant #289 found goal setting “…*quite interesting…because I don’t actually do goal setting…I’m probably getting better at that.”* Participants also discussed learning of more practical skills. For example, participant #337 spoke about use of a facilitative delivery style*:*

*“I think that’s certainly made me think about it differently, starting to think, “Well, these are the exercises we’d maybe like to do, but it’s up to you to choose where to start,” and I like that side of it.”*

Participants reported varying degrees of self-efficacy to deliver the BeST programme. While two participants’ felt very confident to deliver BeST, *“I think having had the training in it…I felt much more confident to deliver it”* (#258), the majority were less confident, particularly around unfamiliar topics, such as delivering the initial individual session *“…we don’t really feel confident doing the individual session because it’s so different…”* (#289).

Of the participants that discussed their attitudes and beliefs towards the management of LBP, the majority reflected biomedical attitudes: *“…I don’t necessarily think that we can just put them straight into that group…because obviously there’s going to be lots of muscle dysfunctions and joint stiffness…”* #226.

#### Therapist behaviour

Participants were anxious about transferring new knowledge and skills to practice, particularly for cognitive-behavioural topics, such as thoughts and feelings. For example, discussing this topic, participant #289 said: *“…it was obvious I was rubbish at it…and even once I knew I was wrong, I couldn’t necessarily see why.”*

## Discussion

### Interpretation of results

This is the first study to explore an online method to build competency in physiotherapists’ use of a CB approach. Mixed methodology enabled us to not only quantify how iBeST performed across a range of learning outcomes; it also provided insight into contextual and unanticipated experiences, captured through interviews. This study did not find large or important differences in outcomes of knowledge and clinical skills, thus suggesting that iBeST may provide sufficient knowledge and skills training to deliver the BeST programme. However, we did observe large changes in attitudes towards the management of LBP among participants in the workshop group, which were not replicated by those trained with iBeST. Importantly, while participants that trained with iBeST were not as highly satisfied as those in the workshop, the majority were still satisfied and found the training method acceptable, providing evidence for the continued use of iBeST. Through integration of qualitative and quantitative methods, we identified strategies that could enhance future versions of iBeST. In particular, we identified strategies that may help to (i) improve user satisfaction and engagement, (ii) improve self-efficacy to use a CB approach, and (iii) achieve a greater change in attitudes and beliefs similar to that observed in the face-to-face workshop.

### Findings in relation to the literature

The similar results on knowledge and skills between both training methods are in line with the largest systematic review to date in this area [13], which reported that online methods were equally effective to alternate forms of training for these outcomes across different health care professions and settings. Further, there has been a growing body of evidence advocating online training as a comparable, if not superior, method for delivering training to health professionals in complex interventions. For example, Dimeff et al. [35] randomised 150 psychologists to receive either a written manual, an online course, or a face-to-face workshop in Dialectical Behaviour Therapy. The online training resulted in a statistically significant greater gain in knowledge, with no other difference between the online and face-to-face training, and with both of these methods outperforming the manual. Similarly, Maloney et al. [36] evaluated the effectiveness of online versus face-to-face training for the prescription of falls prevention exercise. They classified this as a complex intervention since it incorporated a broad range of practical skills including decision making, hands-on skills, and high-level communication. They randomised a range of health care professionals (*n* = 135) to the two training arms, and found no differences between the two groups, reporting comparable satisfaction, knowledge scores, and self-reported changes in clinical practice.

With regards to learner engagement, literature suggests that more engaged users achieve better learning outcomes [37]. Our pragmatic division of engagement scores also support this basic learning principle. This study expands current knowledge concerning engagement of users, identifying barriers to engagement such as prioritising training (due to increased flexibility) and self-discipline that are applicable to all authors of online materials. In addition to the influence of these identified factors on engagement, the presence of face-to-face training itself may have influenced engagement and satisfaction with iBeST among learners perceiving the workshop to be more useful [29].

### Limitations

This feasibility study is limited by its small sample, particularly for the assessment of clinical skills, and while the statistical results give an indication of potential effect, the study was not powered to determine effectiveness. Additionally, the small sample size makes it challenging to confirm that randomisation resulted in a random distribution across the groups. However, the randomisation procedure was implemented without difficulty and there was no evidence of subversion. Therefore, the quantitative results should be interpreted cautiously in the context that this feasibility study provides initial data from which to enhance and further evaluate iBeST. Another limitation is that the assessment of clinical skills from audio recordings was conducted by a unblinded rater. However, to reduce potential bias, 25 % of recordings were doubly assessed using a blinded rater. Similar to other learning courses, the knowledge test was bespoke and, while we assessed face validity with experts in the field, other clinometric properties are not known. Lastly, uptake of the BeST programme in clinical practice was low in both groups, indicating that both forms of training were not adequate to support use in practice in their current format. However, since implementation was not specifically targeted and was simply observed, we think it was encouraging to see at least a third of participants in both groups adopting it in clinical practice.

With respect to the interview study, we used a volunteer sample that may not have been fully representative of the whole sample as our data suggested that the included volunteers were more engaged with the online programme than those declining to take part. Additionally, all interviewees were female, reducing the representativeness of the sample.

### Implications and future work

The results from this evaluation suggest that online learning is a feasible and acceptable method for providing training on a large scale in evidence-based complex interventions such as the BeST programme. The mixed methods employed in this study identified areas where iBeST needs to be enhanced, and a relevant user group (physiotherapists) provided information on how it could be improved. In particular, qualitative data identified that expansion of education on specific cognitive-behavioural elements and inclusion of more skills-based practice would improve therapists’ self-efficacy and satisfaction. This finding is consistent with one of the only studies to explore physiotherapists’ use of a CB approach in clinical practice, which found that despite a 4-day face-to-face workshop with ongoing mentorship, physiotherapists had difficulties adopting some cognitive behavioural aspects of a CB approach programme for patients with osteoarthritis [38]. These results highlight the need to improve upon current strategies for teaching and supporting physiotherapists to deliver a CB approach regardless of training method.

Future work should refine and further evaluate iBeST based on feedback from participants in this study. Additionally, evidence-based implementation strategies should be explored to ascertain how iBeST can be enhanced to support implementation and maximise its impact on clinical practice.

## Conclusion

This evaluation suggests that iBeST is a viable option for widespread dissemination of the BeST training and programme materials that warrants further refinement and evaluation. Using mixed methods enabled us to identify and explore key areas of iBeST that need enhancing. In recognition of the urgent need to manage LBP with CB approach, iBeST provides a promising avenue for building competency on a large scale in physiotherapists’ management of LBP to tackle this growing public health concern.

## Abbreviations

CB, cognitive behavioural; CI, confidence interval; Hr, hours; LBP, low back pain; MD, mean difference; Mm, minutes; NHS, National Health Service; NICE, National Institute of Health and Care Excellence; PABS-PT, Pain, Attitudes, and Beliefs scale for Physiotherapists; RCT, randomised controlled trial; UK, United Kingdom

## Additional files

Additional file 1: Randomised controlled trial baseline dataset.pdf. Baseline quantitative dataset for participants in the randomised controlled trial. (PDF 75 kb) Additional file 2: Randomised controlled trial follow-up dataset.pdf. Follow-up quantitative dataset for participants in the randomised controlled trial. (PDF 72 kb)
