# Supplementary material for: Using mixed methods evaluation to assess the feasibility of online clinical training in evidence based interventions: a case study of cognitive behavioural treatment for low back pain
Source: BMC Med Educ. 2016 Jun 18;16:163. doi: 10.1186/s12909-016-0683-4 (PMC4912756; doi:10.1186/s12909-016-0683-4)
Supplement: Additional file 1: — Randomised controlled trial baseline dataset.pdf. Baseline quantitative dataset for participants in the randomised controlled trial. (PDF 75 kb) [file 12909_2016_683_MOESM1_ESM.pdf]

ID	Age	Years Worked	Computer access	Location of access	Preference	Prior experience	Type of experience	Group	Centre	PABS-PT Bio	PABS-PT Psy	Gender	Delivered BeST	Preference Vs Allocation	Self efficacy assessment	Self efficacy group	Withdrawn
239	26-35 years	10	Daily	Work and Home	No preference	None	None	Online	UHCW	30	24	Female	No	No preference			Withdrew
208	26-35 years	6	Daily	Work only	Face preference	s previous experien	Informal	Online	SWFT	25	18	Female	Yes	Not received preference	9.7	8	Completed
289	56-65 years	35	Daily	Work and Home	No preference	s previous experien	Formal	Online	UHCW	30	27	Female	Yes	No preference	8.9	8	Completed
243	36-45 years	15	2-3 times / week	Work and Home	Face preference	s previous experien	Informal	Online	SWFT	33	22	Female	No	Not received preference	4.4	8.4	Completed
258	26-35 years	8	3-4 times / week	Work only	Face preference	s previous experien	Formal	Online	Oxford	26	22	Female	No	Not received preference	5.1	7	Completed
337	46-55 years	31	3-4 times / week	Work and Home	Face preference	s previous experien	Formal	Online	SWFT	29	25	Female	Yes	Not received preference	5.9	6.5	Completed
197	36-45 years	21	Daily	Work and Home	No preference	s previous experien	Formal	Online	SWFT	30	27	Female	No	No preference	6.1	6.5	Completed
257	36-45 years	22	Daily	Work and Home	Online preference	None	None	Online	UHCW	33	28	Female	Yes	Received preference	4	6	Completed
350	26-35 years	6	Daily	Work only	Face preference	s previous experien	Informal	Online	ROH	30	24	Male	No	Not received preference	4.3	4.7	Completed
226	46-55 years	31	Daily	Work only	Face preference	s previous experien	Informal	Online	Worcs	17	26	Female	Yes	Not received preference	6.5	7.3	Completed
366	18-25 years	2	Daily	Work and Home	No preference	s previous experien	Formal	Online	HOE	27	24	Female	No	No preference	5.5	5.3	Completed
288	26-35 years	10	2-3 times / week	Work only	Face preference	None	None	Online	HOE	33	21	Female	No	Not received preference	5.1	5.2	Completed
209	18-25 years	4	Daily	Work and Home	Online preference	s previous experien	Informal	Online	HOE	30	21	Female	No	Received preference	6	6.7	Completed
232	26-35 years	6	Daily	Work and Home	Online preference	None	None	Online	HOE	30	22	Male	No	Received preference	1.5	1.2	Completed
276	36-45 years	18	Daily	Work and Home	Online preference	s previous experien	Informal	Online	HOE	23	19	Female	No	Received preference	6.6	6.5	Completed
281	26-35 years	3	Daily	Work only	No preference	s previous experien	Formal	Online	HOE	34	23	Male	No	No preference	5.2	5.7	Completed
205	26-35 years	7	Daily	Work and Home	No preference	s previous experien	Informal	Face to face	SWFT	49	21	Female	Yes	No preference	6.6	6.9	Completed
247	56-65 years	30	Daily	Work and Home	Face preference	s previous experien	Formal	Face to face	SWFT	37	22	Female	Yes	Received preference	4.4	1	Completed
360	26-35 years	3	Daily	Work only	Face preference	s previous experien	Informal	Face to face	SWFT	29	23	Male	No	Received preference			Withdrew
170	26-35 years	10	Daily	Work and Home	No preference	None	None	Face to face	HOE	29	20	Male	No	No preference			Withdrew
283	26-35 years	11	Daily	Work and Home	No preference	s previous experien	Informal	Face to face	UHCW	20	29	Female	Yes	No preference	8.2	8.3	Completed
347	26-35 years	7	Daily	Work and Home	Face preference	None	None	Face to face	Oxford	24	20	Female	No	Received preference	8.9	6.6	Completed
199	36-45 years	6	Daily	Work only	No preference	None	None	Face to face	Oxford	37	25	Male	No	No preference	8.4	9.6	Completed
352	18-25 years	2	4-5 times / week	Work only	No preference	s previous experien	Informal	Face to face	UHCW	36	20	Female	No	No preference	5.5	1.2	Completed
260	26-35 years	6	Daily	Work and Home	Online preference	s previous experien	Formal	Face to face	SWB	23	26	Male	Yes	Not received preference	6.6	6.8	Completed
238	26-35 years	5	Daily	Work only	No preference	s previous experien	Formal	Face to face	ROH	38	26	Female	No	No preference	7.9	7.7	Completed
278	26-35 years	11	Daily	Work only	Online preference	s previous experien	Informal	Face to face	ROH	39	21	Female	No	Not received preference	8.1	8.7	Completed
302	46-55 years	30	Daily	Work and Home	Face preference	s previous experien	Informal	Face to face	Worcs	36	17	Female	Yes	Received preference	9.7	5.7	Completed
287	36-45 years	16	Daily	Work and Home	No preference	s previous experien	Formal	Face to face	Derby	28	22	Female	Yes	No preference	4.6	4	Completed
236	36-45 years	17	Daily	Work only	Face preference	s previous experien	Informal	Face to face	Derby	40	26	Female	Yes	Received preference	6.4	7.9	Completed
326	26-35 years	5	Daily	Work only	Face preference	s previous experien	Informal	Face to face	HOE	28	22	Female	No	Received preference	7.2	8.2	Completed
345	26-35 years	9	Daily	Work and Home	Face preference	s previous experien	Informal	Face to face	HOE	22	29	Female	No	Received preference			Withdrew
246	36-45 years	8	Daily	Work and Home	No preference	s previous experien	Informal	Face to face	HOE	32	23	Male	No	No preference	7.9	5.3	Completed
273	26-35 years	7	Daily	Work and Home	No preference	s previous experien	Formal	Face to face	HOE	30	22	Male	No	No preference	9.1	7.5	Completed
255	36-45 years	4	Daily	Work and Home	Face preference	s previous experien	Informal	Face to face	HOE	32	28	Male	No	Received preference	8.6	7.8	Completed
