# Supplementary material for: Using mixed methods evaluation to assess the feasibility of online clinical training in evidence based interventions: a case study of cognitive behavioural treatment for low back pain
Source: BMC Med Educ. 2016 Jun 18;16:163. doi: 10.1186/s12909-016-0683-4 (PMC4912756; doi:10.1186/s12909-016-0683-4)
Supplement: Additional file 2: — Randomised controlled trial follow-up dataset.pdf. Follow-up quantitative dataset for participants in the randomised controlled trial. (PDF 72 kb) [file 12909_2016_683_MOESM2_ESM.pdf]

ID	Group	PABS-PT Bio	PABS-PT Psy	Knowledge	Satisfaction	Self efficacy assessment	Self efficacy group	Preference	Allocated to preference	Change_PAB S1	Change_PAB S2	Delivered BeST	CTS_R	Baseline_PA BS-PT Bio	Baseline_PA BS-PT Psy	Engagement	Group_Eng
239	Online training							No preference	Yes					30	24		
366	Online training	27	26	23	Satisfied	5.5	5.3	No preference	Yes	0	2	No		25	18	1	Least engaged
288	Online training	29	26	29	Neither	5.1	5.2	Face to face preference	No	-4	5	No		30	27	2.33	Most engaged
209	Online training	32	17	27	Satisfied	6	6.7	Online preference	Yes	2	-4	No		33	22	1.67	Least engaged
232	Online training	31	21	19.5	Satisfied	1.5	1.2	Online preference	Yes	1	-1	No		26	22	2	Least engaged
276	Online training	26	17	23	Satisfied	6.6	6.5	Online preference	Yes	3	-2	No		29	25	1.67	Least engaged
281	Online training	35	16	26	Neither	5.2	5.7	No preference	Yes	1	-7	No		30	27	1	Least engaged
208	Online training	24	18	26	Satisfied	9.7	8	Face to face preference	No	-1	0	Yes	1.79	33	28	2.33	Most engaged
289	Online training	34	31	27.5	Neither	8.9	8	No preference	Yes	4	4	Yes	1.93	30	24	2.33	Most engaged
243	Online training	25	20	29	Satisfied	4.4	8.4	Face to face preference	No	-8	-2	No		17	26	2.33	Most engaged
258	Online training	27	23	24.5	Unsatisfied	5.1	7	Face to face preference	No	1	1	No		27	24	2.67	Most engaged
337	Online training	35	20	27	Very Satisfied	5.9	6.5	Face to face preference	No	6	-5	Yes	2	33	21	2.67	Most engaged
197	Online training	25	25	27	Satisfied	6.1	6.5	No preference	Yes	-5	-2	No		30	21	2.33	Most engaged
257	Online training	21	32	29.5	Satisfied	4	6	Online preference	Yes	-12	4	Yes	2.13	30	22	2.67	Most engaged
226	Online training	17	26	30	Satisfied	6.5	7.3	Face to face preference	No	0	0	Yes	1.67	23	19	3	Most engaged
350	Online training			29.5	Satisfied	4.3	4.7	Face to face preference	No			No		34	23	2	Least engaged
326	Face to face training	19	30	23.5	Very Satisfied	7.2	8.2	Face to face preference	Yes	-9	8	No		49	21		
345	Face to face training							Face to face preference	Yes					37	22		
246	Face to face training	22	24	23.5	Very Satisfied	7.9	5.3	No preference	Yes	-10	1	No		29	23		
273	Face to face training	24	26	21	Very Satisfied	9.1	7.5	No preference	Yes	-6	4	No		29	20		
255	Face to face training	24	14	20	Very Satisfied	8.6	7.8	Face to face preference	Yes	-8	-14	No		20	29		
205	Face to face training	39	22	24.5	Very Satisfied	6.6	6.9	No preference	Yes	-10	1	Yes	2.38	24	20		
247	Face to face training	30	25	21	Satisfied	4.4	1	Face to face preference	Yes	-7	3	Yes	2.36	37	25		
360	Face to face training							Face to face preference	Yes					36	20		
170	Face to face training							No preference	Yes					23	26		
283	Face to face training	16	30	27.5	Very Satisfied	8.2	8.3	No preference	Yes	-4	1	Yes	1.93	38	26		
347	Face to face training	24	25	29.5	Very Satisfied	8.9	6.6	Face to face preference	Yes	0	5	No		39	21		
199	Face to face training	21	27	29	Very Satisfied	8.4	9.6	No preference	Yes	-16	2	No		36	17		
352	Face to face training	33	22	28.5	Very Satisfied	5.5	1.2	No preference	Yes	-3	2	No		28	22		
260	Face to face training	16	25	26	Satisfied	6.6	6.8	Online preference	No	-7	-1	Yes	1.79	40	26		
238	Face to face training	30	29	28.5	Satisfied	7.9	7.7	No preference	Yes	-8	3	No		28	22		
278	Face to face training	35	27	30.5	Very Satisfied	8.1	8.7	Online preference	No	-4	6	No		22	29		
302	Face to face training	26	30	23.5	Very Satisfied	9.7	5.7	Face to face preference	Yes	-10	13	Yes	2.5	32	23		
287	Face to face training	17	30	26.5	Satisfied	4.6	4	No preference	Yes	-11	8	Yes	1.64	30	22		
236	Face to face training	26	29	25.5	Satisfied	6.4	7.9	Face to face preference	Yes	-14	3	Yes	1.93	32	28		
